# Molecular characterization of D variants in Brazilian blood donors and their association with RhCE and Duffy phenotypes

**DOI:** 10.1111/vox.70283

**Published:** 2026-05-08

**Authors:** Thamy C. S. Silva, Marcia R. Dezan, Sidneia Costa, Beatriz C. Girardo, Bruno R. Cruz, Karen Ziza, Dante M. Langhi, José O. Bordin, Lilian Castilho, Carla L. Dinardo

**Affiliations:** ^1^ Department of Clinical and Experimental Oncology Federal University of São Paulo São Paulo Brazil; ^2^ Department of Pharmaceutical Sciences Federal University of São Paulo Diadema Brazil; ^3^ Fundação Pró‐Sangue São Paulo Brazil; ^4^ Red Cell and Molecular Blood Group Laboratory Hemocentro‐UNICAMP Campinas São Paulo Brazil

**Keywords:** admixed population, D variants, genotyping, immunohaematology, RHD

## Abstract

**Background and Objectives:**

Serological classification of RhD‐negative and weak D is often insufficient in admixed populations, where diverse *RHD* variants have clinical implications. In Brazil's highly admixed population, RhD interpretation and donor–recipient matching are particularly challenging. We used Duffy phenotyping to support ancestry inference and contextualize Rh variants. Understanding *RHD* alleles and their associations with RhCE and Duffy is essential for transfusion safety. Here, we characterize *RHD* variants in Brazilian donors and relate them to RhCE and Duffy phenotypes across regions.

**Materials and Methods:**

We analysed 321 blood donor samples with either weak D expression (*n* = 295) or a D‐negative phenotype with C and/or E antigen positivity (*n* = 26). RhD, RhCE and Duffy phenotyping was performed, followed by *RHD*, *RHCE* and *FY* genotyping using multiplex polymerase chain reaction with sequence‐specific primers (PCR, PCR‐SSP) and real‐time PCR. Samples unresolved by targeted assays underwent sequencing.

**Results:**

Among weak D donors, 88% carried known *RHD* variants, with weak D types 1, 2 and 3 accounting for 67% of cases. Partial D alleles such as *DAR*, *DAU4* and weak partial 11 were identified, particularly among donors with Fy(a−b−) and Fy(a+b−) phenotypes, often associated with RHCE*733G. In the D‐negative group, 50% of samples harboured non‐functional or hybrid *RHD* alleles, including RHD*03N.01/RHD*08N.01 and RHD*08N.01/RHD*01N.01, linked to the r's haplotype and RHCE*ceS allele.

**Conclusion:**

This study reveals the wide spectrum of *RHD* variants in an admixed population and underscores the importance of integrating molecular genotyping and phenotype associations to improve transfusion safety and guide Rh immunoprophylaxis.

**Highlights**

- Integration of serology, *RHD/RHCE* genotyping and Duffy phenotyping clarifies weak versus partial D in an admixed donor population.
- Regional *RHD* variant profiles align with RhCE backgrounds and Duffy markers, reflecting Brazil's admixture and aiding phenotype interpretation.
- A genotype‐informed approach supports safer RhD classification, guiding donor–recipient matching and Rh prophylaxis when resources are limited.

## INTRODUCTION

The molecular characterization of blood group antigens improves transfusion safety and clinical decision making, particularly in admixed populations. Within this context, the Rh system is among the most clinically significant because of its extensive allelic diversity in *RHD* and *RHCE*, which drives weak and partial D phenotypes and contributes to alloimmunization [1]. Weak D types 1–3, common in individuals of European descent, are typically non‐immunogenic, whereas partial D alleles, more frequent among individuals with African ancestry, may lack specific D epitopes and can elicit anti‐D [1, 2].

We additionally considered the Duffy system (*ACKR1*) as an ancestry‐informative marker, not to claim Duffy is among the most polymorphic system, but because the Fy(a−b−) phenotype driven by FY*02N.01 is strongly enriched in African ancestry. In admixed settings, Duffy can help contextualize the background in which particular *RHD* and *RHCE* alleles arise, offering practical value when molecular testing resources must be triaged [3, 4].

Brazil is one of the most genetically admixed countries worldwide, with regional heterogeneity reflecting varying European, African and Indigenous contributions. This admixture shapes blood group allele distributions across regions and influences the prevalence of weak and partial D alleles and *RHCE* haplotypes. Consequently, integrating ancestry‐informative phenotypes with *RHD*/*RHCE* genotyping may enhance the interpretation of discrepant RhD serology and inform donor–recipient matching strategies in Brazil.

Here, we characterize *RHD* variant alleles in Brazilian blood donors, relate them to RhCE backgrounds and Duffy phenotypes across regions and, to our knowledge, provide the first multi‐regional, donor‐based integration from Brazil. We also benchmark our findings against international cohorts to place Brazilian allele distributions in a global context and to inform genotype‐aware transfusion strategies in admixed settings.

## MATERIALS AND METHODS

### Blood donor samples

The study was conducted at the Federal University of São Paulo in collaboration with Fundação Pró‐Sangue and Imunolab Laboratory, both located in São Paulo, Brazil. Between July 2019 and July 2020, Imunolab processed 311,998 blood donor samples from four major regions of Brazil (Northeast, Southeast, South and Central‐West). Of the 311,998 blood donor samples processed during the study period, 562 (0.18%) were classified as weak D. Among these, 295 (52.5%) were included in the present study based on residual sample availability, sufficient volume for DNA extraction, sample integrity after storage/transport and laboratory capacity for molecular testing within the predefined study resources. Inclusion was consecutive according to availability and was not based on serological reaction strength or suspected genotype. Additionally, 486 samples from donors serologically typed as RhD‐negative but expressing C and/or E antigens were screened. Following molecular genotyping, 26 of these samples were found to carry the *RHD* gene and were included in the study. In total, 321 samples were analysed, divided into two groups:

- Group 1 (weak D): 295 samples with weak serological expression of the D antigen;
- Group 2 (RhD‐negative with C and/or E positive): 26 samples with serologically negative RhD typing, negative confirmation for weak D but RhC and/or RhE positive and molecular detection of the *RHD* gene.

### Serologic tests

Samples in Group 1 (weak D) were first tested on the NEO platform (Werfen/Immucor) using an immunoglobulin M + immunoglobulin G anti‐D blend (clones D175‐2 and D415‐1E4) and the anti‐D rapid reagent (clone RUM‐1, Immucor). The analyser acquires an image of each well and applies multi‐feature image analysis to generate a numeric reaction score on a 0–100 scale for each result; the instrument software then maps this score to the final interpretation according to assay‐specific cut‐offs validated in our laboratory. For study enrolment, we classified samples as showing low/weak RhD expression when the NEO reaction score was ≤80. For interpretive context, published analyses have shown a practical correlation between the NEO score and conventional reaction grades (0–4+): in RhD testing, scores >80 generally align with ~4+ and typical score ranges have been reported for variant categories, weak D ~ 10–90 and partial D ~ 60–99, supporting the use of the ≤80 threshold to capture low/weak expression [5]. Confirmation of weak D was performed with the Capture‐R Select solid‐phase assay (Immucor) using the same antibody blend. All samples were negative in the direct antiglobulin test (DAT).

Samples in Group 2 (RhD‐negative with RhC and/or RhE positive) followed the same initial serological protocol, with RhD typing scores ≤80 and negative results in the weak D confirmation test by solid‐phase assay. All these samples expressed RhC and/or RhE antigens. RhCE phenotyping was carried out by microplate haemagglutination using monoclonal anti‐C antibodies (clones MS‐24 and MS‐273) and anti‐E antibodies (clones MS‐80 and MS‐258).

Given the highly admixed nature of the Brazilian population and the absence of self‐reported ancestry data, Duffy phenotyping was included as an additional ancestry‐informative marker. Duffy typing was performed using gel column agglutination (DG Gel Liss/Coombs cards, Grifols) with anti‐Fya antibodies (clones DG‐FYA‐02 or P3TIM) and polyclonal anti‐Fyb antibodies (Fresenius‐Kabi).

### DNA extraction and molecular testing

Genomic DNA was extracted from nucleated cells using TRI reagent (a monophasic solution of phenol and guanidine isothiocyanate) (Sigma‐Aldrich, USA), following the manufacturer's instructions. Concentration and purity were assessed with a NanoDrop ND‐1000 spectrophotometer (Thermo Fisher Scientific).

Weak D samples (Group 1) were screened by multiplex PCR for common *RHD* variants using previously described primers [6]. D‐negative samples with C and/or E positivity (Group 2) were tested for *RHD* intron 4 and exon 7 using multiplex PCR in pools of 10 [7]. Samples positive with intron 4 and exon 7 were further analysed for *RHD* zygosity via allele‐specific PCR targeting hybrid Rhesus‐box regions [8, 9]. Weak D type 1 was identified by PCR followed by ApaL I restriction digestion [10]. Additional PCR‐SSP assays targeted the RHD*1227A and RHD*602c.G>C single nucleotide polymorphisms, associated with partial D alleles (*DAR*, *DAU*) [11, 12]. A PCR multiplex was used to identify RHD*D‐CE(4–7)‐D [13]. A subset was genotyped for RHCE*733G and RHCE*1006T, which define variant RhCE phenotypes [14].

For donors with Fy(a−b−) or Fy(a+b−) phenotypes, FY*02N.01 detection was performed by real‐time PCR using custom Thermo Fisher assays (rs12075 and rs2814778) targeting FY*c.125A>G and GATA*c.‐67T>C polymorphisms. Reactions were run on a QuantStudio 5 system, with fluorescence monitored from cycle 15 to distinguish genotypes.

Descriptive frequencies were calculated as proportions and 95% confidence intervals for carrier frequencies in the Brazilian cohort were estimated using the Wilson binomial method.

## RESULTS

### Results—Group 1 (weak D samples)

Among the 295 Group 1 samples, genotyping revealed that 260 (88%) carried at least one allele associated with altered RhD antigen expression. The most common variants were weak D types 1, 2 and 3, accounting for 173 samples (67%). Weak D type 2 was the most prevalent, found in 116 samples (39%), followed by weak D type 1 in 45 samples (15%) and weak D type 3 in 12 samples (4%). Other detected variants included weak D type 38 in 34 samples (12%) and weak D type 15 in 3 samples (1%). Additionally, partial D alleles were identified in 50 samples (19%), including weak partial D 11 in 21 samples (7%) and *DAR* alleles in 11 samples (4%), of which 4 were classified as RHD*DAR2.01 and 7 as RHD*DAR1.02 (also known as weak D type 4.2.2). As these two differ only by the single nucleotide variant c.697G>C, they were collectively referred to as RHD*DAR. The RHD*DAU4 allele was identified in 18 samples (6%). A total of 35 samples (12%) were not resolved by the targeted molecular workflow. In these cases, none of the screened variants was detected, and full‐gene sequencing was not feasible in all samples because of limited residual material.

*RHCE* phenotypes were: C+E− 43%, C−E+ 42%, C+E+ 2%, C−E− 11%. Expected linkages were confirmed: weak D types 1/3/38 with C+E−; weak D types 2/15 with C−E+; DAR and DAU4 with C−E− (Table 1).

**TABLE 1:** Molecular characterization of weak and partial D variants in serologically RhD‐positive blood donors with reduced D expression (Group 1).

D variant	*N* total	ISBT nomenclature	RhCE	*N*	Duffy	*N*	Region	*N*
Weak D type 1	45	RHD*weak D type 1	C+E−	43	Fy(a−b–)	0	Southeast	17
			C−E+	2	Fy(a+b−)	10	Northeast	7
			C+E+	0	Fy(a+b+)	22	Central‐West Brazil	11
			C−E−	0	Fy(a−b+)	13	South	10
			Total	45		45		45
Weak D type 2	116	RHD*weak D type 2	C+E−	0	Fy(a−b−)	11	Southeast	36
			C−E+	114	Fy(a+b−)	22	Northeast	22
			C+E+	2	Fy(a+b+)	36	Central‐West Brazil	38
			C−E−	0	Fy(a−b+)	47	South	20
			Total	116		116		116
Weak D type 3	12	RHD*weak D type 3	C+E−	12	Fy(a−b−)	1	Southeast	7
			C−E+	0	Fy(a+b−)	5	Northeast	1
			C+E+	0	Fy(a+b+)	5	Central‐West Brazil	4
			C−E−	0	Fy(a−b+)	1	South	0
			Total	12		12		12
Weak D type 38	34	RHD*weak D type 38	C+E−	34	Fy(a−b−)	0	Southeast	12
			C−E+	0	Fy(a+b−)	10	Northeast	7
			C+E+	0	Fy(a+b+)	12	Central‐West Brazil	5
			C−E−	0	Fy(a−b+)	12	South	10
			Total	34		34		34
Weak D type 11	21	RHD*weak partial 11	C+E−	18	Fy(a−b−)	4	Southeast	9
			C−E+	1	Fy(a+b−)	7	Northeast	1
			C+E+	2	Fy(a+b+)	6	Central‐West Brazil	4
			C−E−	0	Fy(a−b+)	4	South	7
			Total	21		21		21
Weak D type 15	3	RHD*weak partial 15	C+E−	0	Fy(a−b−)	0	Southeast	0
			C−E+	3	Fy(a+b−)	3	Northeast	0
			C+E+	0	Fy(a+b+)	0	Central‐West Brazil	0
			C−E−	0	Fy(a−b+)	0	South	3
			Total	3		3		3
DAR 1.2 (weak D 4.2.2) or DAR 2.1	11	RHD*09.01.02 or RHD*09.02.01	C+E−	0	Fy(a−b−)	2	Southeast	5
			C−E+	0	Fy(a+b−)	4	Northeast	2
			C+E+	0	Fy(a+b+)	1	Central‐West Brazil	4
			C−E−	11	Fy(a−b+)	4	South	0
			Total	11		11		11
DAU4	18	RHD*10.05	C+E−	0	Fy(a−b−)	0	Southeast	5
			C−E+	0	Fy(a+b−)	7	Northeast	7
			C+E+	0	Fy(a+b+)	8	Central‐West Brazil	5
			C−E−	18	Fy(a−b+)	3	South	1
			Total	18		18		18
Technically unresolved (no targeted variant detected)	35	RHD*01.01	C+E−	21	Fy(a−b−)	11	Southeast	8
			C−E+	5	Fy(a+b−)	8	Northeast	14
			C+E+	1	Fy(a+b+)	5	Central‐West Brazil	7
			C−E−	3	Fy(a−b+)	11	South	6
			Total	35		35		35

*Note*: RhCE phenotype, Duffy phenotype and geographic region are shown as distributions within each RhD variant category. For each variant, subgroup counts sum to the total number of samples in that category, as indicated in the ‘Total’ row.

Abbreviation: ISBT, international society of blood transfusion.

Among samples carrying the RHD*DAR allele, six were genotyped for the RHCE*733 polymorphism: two (33%) were heterozygous (RHCE*733C/G) and four (67%) were homozygous for the variant (RHCE*733G/G). Of the RHD*DAU4 positive samples, seven were genotyped: five (71%) were homozygous for RHCE*733C, one (14%) was heterozygous and one (14%) was homozygous for RHCE*733G.

Duffy phenotyping and *FY* promote: Duffy phenotypes were Fy(a−b−) 10% (29/295), Fy(a−b+) 32% (95/295), Fy(a+b−) 26% (76/295) and Fy(a+b+) 32% (95/295). Among Fy(a−b−) donors, the most frequent *RHD* associations were weak D type 2 (38%), followed by weak partial 11 (14%), RHD*DAR (7%) and weak D type 3 (3%). The Fy(a+b−) group showed a broader mix, led by weak D type 2 (29%), with weak D types 1 and 38 (each 13%), and smaller contributions from weak partial 11 and RHD*DAU4 (each 9%), weak D type 3 (7%), RHD*DAR (5%) and weak D type 15 (4%) (Table 1). In the subset selected for *FY* promoter genotyping (*n* = 105; Fy(a−b−) or Fy(a+b−)), conclusive results were obtained for 101 samples, whereas 4 samples yielded inconclusive results. Among samples with conclusive genotyping, FY*02N.01 was homozygous in 26% (26/101), heterozygous in 36% (36/101) and wild‐type in 39% (39/101). Across *FY* promoter–genotype groups, weak D type 2 was the most common *RHD* allele.

**FIGURE 1:**
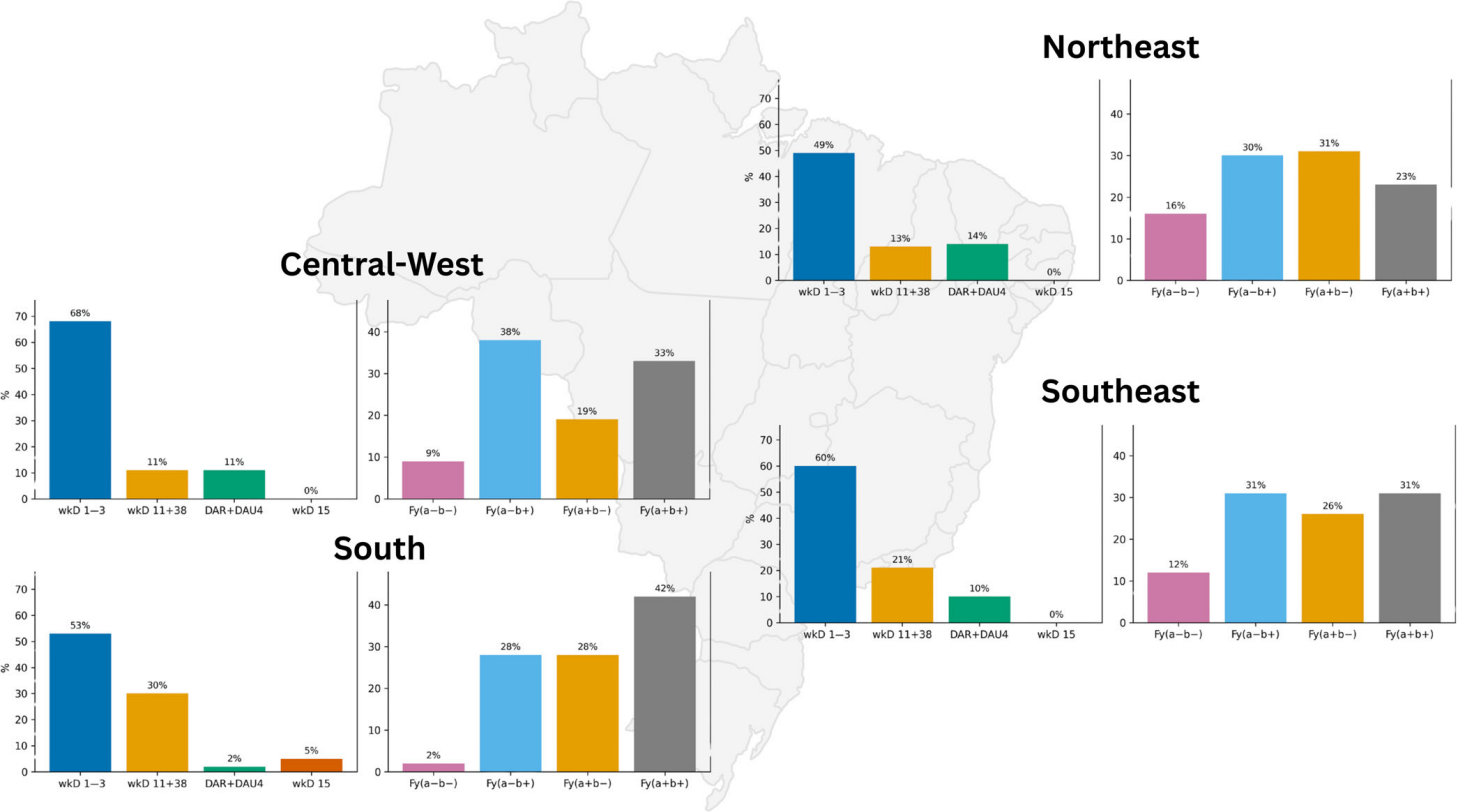
Regional distributions of RHD variant groups and Duffy phenotypes in Brazilian donors. Bars show the percentage of sampled donors within each region (Southeast, Northeast, Central‐West, South). wkD = weak D; categories for RHD variant groups are wkD 1–3 (weak D types 1–3), wkD 11 + 38 (weak partial D type 11 plus weak D type 38), DAR + DAU4 (partial D alleles DAR and DAU4) and wkD 15 (weak D type 15). Duffy phenotypes are shown as Fy(a−b−), Fy(a−b+), Fy(a+b−) and Fy(a+b+). All values are expressed as percent (%) of donors in the respective region. Bars are colour‐coded using a colour‐blind friendly palette.

When stratified by geographic region of Brazil, the distribution of RhD variants and Duffy phenotypes showed expected trends based on known historical admixture. Donors from the Southeast and Northeast exhibited a higher frequency of African‐associated markers, such as the FY*02N.01 allele and *RHD* alleles like RHD*DAR and RHD*DAU4, consistent with the higher African ancestry in these regions. In contrast, donors from the South showed a predominance of weak D types more commonly found in European populations, such as weak D types 1 and 2. Figure 1 summarizes the regional distributions of *RHD* variant groups (weak D 1, 2 and 3, weak D 11 + 38, DAR + DAU4 and weak D 15) and Duffy phenotypes, illustrating higher African‐associated signals in the Southeast/Northeast.

### Results—Group 2 (D‐negative, C and/or E‐positive samples)

A total of 486 D‐negative donor samples expressing the C and/or E antigens were initially screened for the presence of the *RHD* gene using multiplex PCR. Among them, 26 samples (5%) tested positive for *RHD* sequences. Of these 26 *RHD*‐positive samples, 5 (19%) carried the RHD*08N.01/RHD*01N.01 genotype. Although these samples were not genotyped for *RHCE*, serological phenotyping revealed that three expressed the C antigen and two expressed the E antigen. Regarding the Duffy phenotypes, one donor was Fy(a+b−), two were Fy(a−b+) and two were Fy(a+b+).

The RHD*03N.01/RHD*08N.01 genotype was identified in eight samples (31%). All of these donors carried the RHCE*ceS allele (also referred to as RHCE*ceVS.03), indicating the presence of the r's haplotype. This haplotype includes two altered genes: a hybrid RHD*D‐CE‐D gene comprising exons 1, 2, 3, 8, 9 and 10 from *RHD* and exons 4 through 7 from *RHCE*, which encodes D negative and partial C antigen and the RHCE*ceS allele, which defines a complex phenotype characterized by V−, VS+, hrB−, partial c and partial e antigen expression.

Among these eight donors, seven exhibited the RHCE*ceVS.03/RHCE*ce genotype and presented with the serological profile: D−, partial C, c+, e+, V−, VS+ and hrB+. One donor carried the RHCE*ceVS.03/RHCE*Ce genotype and showed the phenotype D−, C+, partial c, e+, V−, VS+ and hrB+. Regarding Duffy phenotypes in this subgroup, three donors (38%) were Fy(a−b−), four (50%) Fy(a+b−), and one (12%) Fy(a−b+).

In the remaining 13 samples (50%), no specific RHD variant included in the targeted testing strategy was identified. Additional extended characterization was not feasible because of limited residual material. These samples tested negative for RHD*Ψ, RHD*03N.01 and RHD*DEL1 alleles.

## DISCUSSION

The Rh blood group system is one of the most polymorphic in humans, with numerous allelic variants in both *RHD* and *RHCE* genes that can lead to altered antigen expression. This genetic variability is a major contributor to serological discrepancies and alloimmunization, particularly in admixed populations. We explored the molecular diversity of RhD variants and their associations with *RhCE* and Duffy phenotypes, emphasizing the value of integrating molecular testing and ancestry‐informative markers to optimize transfusion safety.

Our results revealed that the most frequent D variants identified were weak D types 2, 1 and 38, followed by 3, 11 and 15. These findings are consistent with Brazil's admixed ancestry, reflecting gene flow from European, African and Asian populations [15, 16, 17, 18]. Weak D type 2 is common in Iberian populations, while types 38 and 15, described in European and East Asian cohorts, further support the diverse genetic background of Brazilian donors [18, 19, 20].

Notably, DAR and DAU alleles—widely reported among individuals of African ancestry—were identified in only 10% of the samples. While these alleles are generally considered partial D variants with clinical significance, their lower representation in our cohort may be related to the study inclusion criteria, which focused on samples with reduced serological reactivity (≤3+; automated score ≤80). Previous studies have shown that lower automated RhD scores correlate with reduced antigen density and enrichment for weakly expressed variants, whereas some partial D alleles may present with stronger reactions and therefore fall outside this selection strategy [5]. This approach may therefore have underrepresented stronger D‐partial phenotypes more commonly recognized in alloimmunized patients (D+ with anti‐D). Nevertheless, the identification of RHD*DAR2.1 in weakly reactive samples raises the possibility that even partial D alleles may present with weak antigen expression under certain genetic backgrounds.

Our analysis also confirmed the expected associations between RhD variants and RhCE haplotypes, reinforcing the value of haplotype‐based inference in transfusion medicine, as previously seen in studies with Brazilians [21]. Variants like types 1, 3, 11 and 38 were mainly linked to C+E− haplotypes, while types 2 and 15 were associated with C−E+. RHD*DAR and RHD*DAU4 were exclusively associated with C−E− (R0), consistent with African ancestry [22, 23]. Tables 1 and 2 summarize the associations of RHD variants with RhCE phenotype, Duffy phenotype and geographic region.

**TABLE 2:** Genotypic profiles of serologically RhD‐negative blood donors carrying *RHD* alleles (Group 2).

D variant	*N* total	ISBT nomenclature	RhCE	*N*	Duffy	*N*	Region	*N*
D‐negative (pseudogene)	8	RHD*03N.01/RHD*08N.01	C+E−	8	Fy(a−b−)	3	Southeast	2
			C−E+	0	Fy(a+b−)	4	Northeast	4
			C+E+	0	Fy(a+b+)	0	Central‐West Brazil	2
			C−E−	0	Fy(a−b+)	1	South	0
			Total	8		8		8
D‐negative (pseudogene)	5	RHD*08N.01/Deleted D	C+E−	3	Fy(a−b−)	0	Southeast	3
			C−E+	2	Fy(a+b−)	1	Northeast	1
			C+E+	0	Fy(a+b+)	2	Central‐West Brazil	0
			C−E−	0	Fy(a−b+)	2	South	1
			Total	5		5		5
Technically unresolved (no targeted variant detected)	13	RHD*01N.01	C+E−	11	Fy(a−b−)	1	Southeast	7
			C−E+	12	Fy(a+b−)	3	Northeast	1
			C+E+	0	Fy(a+b+)	2	Central‐West Brazil	1
			C−E−	0	Fy(a−b+)	7	South	4
			Total	13		13		13

*Note*: RhCE phenotype, Duffy phenotype and geographic region are shown as distributions within each RhD variant category. For each variant, subgroup counts sum to the total number of samples in that category, as indicated in the ‘Total’ row.

Abbreviation: ISBT, international society of blood transfusion.

In admixed populations like Brazil, self‐reported ancestry often fails to accurately reflect genetic background relevant to transfusion safety. The Duffy blood group system, especially the Fy(a−b−) phenotype and the FY*02N.01 allele (c.1‐67T>C in the GATA promoter), is a well‐established ancestry‐informative marker associated with African heritage and has been shown in Brazilian cohorts to correlate more closely with African genomic ancestry than self‐declared race in specific clinical settings [24, 25]. The distribution of Duffy phenotypes in this study aligns with patterns of genetic admixture observed in Brazil, where African ancestry is more prominent in regions such as the Southeast, Northeast and North. Although Duffy markers do not fully capture individual genomic ancestry and phenotype–genotype discordances may occur in highly admixed populations, they remain a practical and biologically relevant proxy when genome‐wide ancestry testing is unavailable. When present, such discordances may modestly reduce the precision of ancestry inference and attenuate associations between Duffy background and specific D variant frequencies. Accordingly, in the present study, Duffy phenotyping was used as a supportive population‐level indicator to contextualize D variant backgrounds rather than as a definitive measure of ancestry.

In this study, among donors with the Fy(a−b−) phenotype, the most frequently observed RhD variants were weak D type 2 and partial D types. The relatively low frequency of alleles such as RHD*DAR, which are typically associated with African ancestry, may reflect the study's focus on individuals with reduced RhD expression rather than a broader survey of all D variants. While these findings support the role of Duffy phenotyping as a useful tool in identifying donors with African ancestry, they also point to the complexity of interpreting genotype–phenotype relationships in highly admixed populations. In contexts where molecular testing is not universally available, combining serological D typing discrepancies with Duffy phenotyping remains a pragmatic approach to prioritize samples for genotyping, especially in resource‐limited settings. However, the predictive power of Duffy markers may vary in highly admixed populations such as Brazil, where genotype–phenotype associations may diverge from patterns observed in ancestrally homogeneous groups.

Despite the limitations, Duffy phenotyping and, when feasible, genotyping of ancestry‐informative markers can aid in identifying individuals who may benefit from extended Rh genotyping. Our findings align with previous reports showing that the Fy(a−b−) phenotype is associated with partial *RHD* alleles of African origin, reinforcing its utility as a screening tool in admixed populations presenting with weak or discrepant RhD serology. However, our study also revealed an unexpected pattern: Fy(a−b−) individuals were more frequently associated with weak D type 2 and partial D type 11, alleles not typically prevalent in populations with predominant African ancestry but observed here, likely reflecting the complex ancestry mosaic generated by historical admixture, population stratification or recombination across generations. This atypical association likely reflects the extensive genetic admixture in Brazil, where African, European and Asian ancestries intermingle, resulting in uncommon combinations of Rh and Duffy genotypes. These findings emphasize that while Duffy phenotyping remains a valuable first‐line tool, it should be interpreted with caution and, ideally, complemented by molecular analyses when precise genotype information is required for transfusion safety.

When interpreted alongside previous Brazilian studies, our findings suggest that the distribution of *RHD* variants in Brazil is not uniform but varies according to both study design and the regional admixture background of the sampled donor population [20, 21, 26]. Previous Brazilian reports documented the presence of African‐associated alleles such as DAR among donors with discrepant D typing, whereas our cohort showed a predominance of weak D types 1 and 2 and lower frequencies of DAR/DAU, together with a measurable contribution of weak D types 38 and 11 [26, 27]. This pattern likely reflects, at least in part, differences in inclusion criteria and serological selection, but also the heterogeneous regional composition of Brazilian donor populations. In this context, the value of our analysis lies not only in reporting allele frequencies but also in showing how *RHD* variants segregate with RhCE backgrounds and Duffy phenotypes across regions, providing practical clues for local molecular screening strategies and for building donor inventories better aligned with the needs of transfusion recipients in admixed settings.

These regional differences are clinically plausible and consistent with broader genomic studies demonstrating marked heterogeneity in Brazilian ancestry proportions, with greater European contribution in the South and higher African contribution in the Northeast and parts of the Southeast [28]. Because *RHD* and *RHCE* variant frequencies track ancestral backgrounds, local enrichment of specific alleles would be expected rather than anomalous. For example, African‐associated alleles such as DAR, DAU and ceS‐related haplotypes have been repeatedly reported at higher frequencies in populations of African descent, whereas weak D types 1, 2 and 3 predominate in European populations [23, 29]. Therefore, our findings suggest that Brazilian blood donor inventories should not be interpreted as genetically interchangeable across regions and that region‐specific molecular data may better reflect the true local availability of compatible Rh units.

This issue is particularly relevant for chronically transfused recipients, especially patients with sickle cell disease (SCD), in whom *RH* genetic diversity and *RHCE* variant haplotypes are strongly associated with persistent alloimmunization despite conventional serological Rh matching [30]. Previous studies have shown that recipients of African ancestry may carry altered *RH* alleles not adequately represented in predominantly European‐derived donor pools, leading to partial antigen exposure and antibody formation [14, 23]. In this context, the present study adds practical value by identifying which Brazilian regions may preferentially contribute donors carrying African‐associated or European‐associated *RH* backgrounds. Such information may support targeted donor recruitment, regionally informed molecular screening panels and more efficient allele‐matched transfusion strategies in a highly admixed country. Considering the high risk of alloimmunization in chronically transfused individuals with SCD, a group in which Duffy‐null and partial D phenotypes are common, our findings reinforce the need for extended phenotyping and molecular genotyping in donor selection. As highlighted in previous studies, characterizing *RHD* and *RHCE* alleles in ethnically diverse populations is key to optimizing transfusion compatibility and reducing alloimmunization [30].

In donors serologically classified as RhD‐negative, molecular detection of *RHD* confirms the presence of silent or hybrid alleles that may encode weak or undetectable D antigen expression. This poses a potential risk for sensitization if such individuals are exposed to conventional D‐positive red blood cells, especially in transfusion or obstetric contexts. The predominant allele identified in this group was RHD*03N.01, commonly associated with hybrid *RHD‐RHCE* genes and the r's haplotype. These individuals frequently carry altered *RHCE* alleles, such as *ceS*, which encode partial c, e and VS antigens. This genetic profile, often observed in individuals of African ancestry, is known to produce complex serological patterns that can challenge routine typing, increasing the risk of misclassification and complicating transfusion and pregnancy management [29, 31]. The identification of RHD*Ψ in D‐negative, *RHD*‐positive individuals highlights the molecular diversity behind the D‐negative phenotype. Unlike the complete *RHD* gene deletion common in European ancestry, non‐functional alleles like RHD*Ψ are more frequent in individuals of African descent and may be missed by standard molecular testing, posing challenges for accurate RhD classification [8]. Although these alleles do not encode a functional D antigen, their identification is essential in multiethnic populations to ensure precise RhD interpretation and avoid misinterpretation [32].

These findings emphasize the limitations of serological methods in accurately classifying RhD status in admixed populations. The detection of weak or partial D variants, many with immunogenic potential, underscores the importance of incorporating molecular testing, particularly in donors with discrepant RhD serology or in clinical scenarios where Rh prophylaxis is critical. While variants such as weak D types 1, 2 and 3 are considered non‐immunogenic, partial D alleles like DAR, DAU and weak partial 11 have been linked to alloanti‐D formation and require careful management. In resource‐limited settings, where genotyping is not routinely available, prioritizing D‐negative transfusions for patients with unclear RhD status, especially women of childbearing age and chronically transfused individuals, remains a prudent strategy. The global comparison of D variant frequencies (Table 3) reinforces that allele distribution is highly ancestry‐dependent, highlighting the value of population‐specific data to inform transfusion policies.

**TABLE 3:** Allele frequencies of RhD variant alleles in admixed Brazilian donors and other global populations.

D variant	Brazilian blood donors			Mali	Sub‐Saharan Africa	French Guiana	Tunisia	Nigeria	French Africans	Croatia	Germany	Austria	France	Japan	China	Australia	Canada	Portugal	References
	*N*	Frequency (%)	95% CI	Published allele frequency ranges (%)															
Weak D type 1	45	15.3	11.6%–19.8%							24.9–49.8	31.5–63.0	16.5–33.1	16.1–32.2			22.0–44.0	14.6–29.1	8.1–16.2	[12, 19, 33, 34, 35, 36, 37]
Weak D type 2	116	39.3	33.9%–45.0%							4.75–9.5	10.4–20.8	3.9–7.7	10.9–21.8			12.5–25.0	7.3–14.6	31.8–63.6	[12, 19, 33, 34, 35, 36, 37]
Weak D type 3	12	4.1	2.3%–7.0%							12.3–24.7	5.9–11.9	25–50	1.9–3.8			4.0–8.0	0.9–1.8	7.1–14.1	[12, 19, 33, 34, 35, 36, 37]
Weak D type 38	34	11.5	8.4%–15.5%										0.17–0.35	0.2–0.4					[16, 19]
Weak D type 11	21	7.1	4.7%–10.6%										1.2–24.2			0.5–1.0			[19, 34]
Weak D type 15	3	1.0	0.3%–2.9%										2.9–5.9	8.0–15.9	15.9–31.8	1.0–2.0			[15, 16, 17, 19, 34]
RHD*DAR (cluster)	11	3.7	2.1%–6.5%	1.2–2.4	8.93–17.9		0.28–0.56	0.16–0.33	0.03		0.8–1.6	0.2–3.1	2.1–4.2			1.5–3.0	11.8–23.6	3.0–6.1	[12, 19, 22, 23, 34, 35, 36, 37, 38, 39, 40, 41]
RHD*DAU (cluster)	18	6.1	3.9%–9.4%	5.1–10.2	16.9–33.7	3.3–3.9	0.25–0.50		0.03				0.35–0.7				4.6–9.1		[19, 22, 23, 35, 38, 39, 41, 42]
RHD*01	35	11.9	3.9%–9.4%	27.1–54.1	16.4–32.6	43.3–70.2			0.69										[38, 39, 41, 42]
Total	295																		

*Note*: Frequencies shown are based on previously published studies (references listed). Brazilian frequencies refer to the present study (*n* = 295) and are presented as carrier frequencies with corresponding 95% confidence intervals. Frequencies from other populations are shown as published allele frequency ranges (%) reported in the original studies and are provided for descriptive comparison only, since study design, inclusion criteria and denominators varied across reports. Some variants were grouped into clusters (e.g., DAR and DAU) when individual allele resolution was not available. See text and references for details.

Table 3 presents a comparative overview of the D variant allele frequencies observed in Brazilian donors alongside data from other global populations. Notably, weak D type 2, which was the most common variant in our cohort, shows high frequencies in Iberian and Central European populations, while variants such as RHD*DAR and RHD*DAU are more prevalent in Sub‐Saharan African populations, aligning with their known ancestral origins. These distributions reinforce the complexity of transfusion genetics in Brazil and the importance of considering regional allele prevalence when implementing transfusion protocols and donor screening strategies.

The detection of hybrid alleles such as RHD*03N.01 in serologically D‐negative donors also draws attention to co‐inherited *RHCE* variants, which may encode partial c or e antigens and lack high‐frequency antigens such as hrB. These donors, although not at risk of anti‐D formation, may represent valuable resources for complex transfusion cases, particularly in patients with SCD.

These observations underscore the need for population‐specific donor screening strategies and support a shift towards genotype‐informed transfusion policies, especially in countries with high genetic diversity. In the absence of routine genotyping, targeted molecular testing based on serological discrepancies or ancestry‐related markers can improve transfusion safety and inform obstetric care.

This study has some limitations. Self‐reported ancestry data were unavailable, limiting direct genotype–ancestry correlation. Although Duffy phenotypes and FY*02N.01 were used as proxies, they cannot replace genomic ancestry inference. Not all samples were fully genotyped for *RHD* and *RHCE* due to DNA constraints, which may have led to underdetection of rare alleles. The focus on samples with weak D expression (≤3+) may have excluded some partial D variants and variants must be interpreted cautiously because of limited numbers. In addition, not all weak D samples identified during the study period were available for molecular analysis, as inclusion depended on residual sample availability, sufficient volume, specimen integrity and laboratory testing capacity; therefore, some selection bias cannot be completely excluded, although samples were not selected according to serological strength or suspected genotype. Donors whose results remained unresolved (*n* = 13 and *n* = 35) will be recontacted, where feasible, for repeat sampling and additional molecular testing in a future phase of the study, with the aim of clarifying the underlying alleles.

In summary, our findings highlight the molecular diversity of RhD variants in an admixed population and reinforce the value of integrating serological, molecular and ancestry‐informative data to improve transfusion safety. Targeted genotyping in donors with discrepant serology or ancestry markers may optimize blood‐matching strategies and reduce the risk of alloimmunization, especially in vulnerable patient groups.

## CONFLICT OF INTEREST STATEMENT

The authors declare no conflicts of interest.

## Data Availability

The data that support the findings of this study are available on request from the corresponding author. The data are not publicly available due to privacy or ethical restrictions.
